# Mental disorders and suicide risk in emerging adulthood: the 1993 Pelotas birth cohort

**DOI:** 10.11606/s1518-8787.20190530012356

**Published:** 2019-10-16

**Authors:** Ana Paula Gomes, Ana Luiza G. Soares, Christian Kieling, Luis Augusto Rohde, Helen Gonçalves

**Affiliations:** I Universidade Federal de Pelotas. Faculdade de Medicina. Programa de Pós-Graduação em Epidemiologia. Pelotas, RS, Brasil; II University of Bristol. Population Health Sciences. Bristol Medical School. Bristol, United Kingdom; III Universidade Federal do Rio Grande do Sul. Faculdade de Medicina. Departamento de Psiquiatria. Porto Alegre, RS, Brasil; IV Hospital de Clínicas de Porto Alegre. Serviço de Psiquiatria da Infância e da Adolescência. Porto Alegre, RS, Brasil

**Keywords:** Young Adult, Mental Disorders, epidemiology, Suicide, Risk Factors, Mental Health

## Abstract

**OBJECTIVE:**

To assess the prevalence of some mental disorders and suicide risk, and the association between them in youths.

**METHODS:**

Data from the 1993 Pelotas Birth Cohort (Brazil) was used. The prevalence of mental disorders at 22 years [major depressive disorder (MDD), generalized anxiety disorder (GAD), social anxiety disorder (SAD), attention-deficit/ hyperactivity disorder (ADHD), bipolar disorders type 1 and 2 (BD1; BD2), post-traumatic stress disorder (PTSD), and antisocial personality disorder (APD)] and of suicide risk were assessed using the Mini International Neuropsychiatric Interview (n = 3,781). Comorbidity between disorders was also assessed. Association of each mental disorder and the number of disorders with suicide risk was assessed using Poisson regression.

**RESULTS:**

The prevalence of any mental disorder was 19.1% (95%CI 17.8–20.3), and GAD was the most prevalent (10.4%; 95%CI 9.5–11.4). The prevalence of current suicide risk was 8.8% (95%CI 5.9–9.7). All disorders (except APD) and the suicide risk were higher among women. Mental disorders were associated with a higher suicide risk, with the highest risks being observed for MDD (RR = 5.6; 95%CI 4.1–7.8) and PTSD (RR = 5.0; 95%CI 3.9–6.3). The higher the number of co-occurring mental disorders, the higher the risk of suicide.

**CONCLUSIONS:**

Our findings showed that about 20% of the youths had at least one mental disorder. However, this prevalence is underestimated since other relevant mental disorders were not assessed. Mental disorders were associated with higher suicide risk, especially the comorbidity between them.

## INTRODUCTION

Emerging adulthood (18–29 years) is a period of many life changes and heightened instability, which can increase the risk of mental disorders^1^ . Although most mental disorders have their onset in childhood or adolescence, more severe and chronic disorders such as personality disorders, mood disorders (depression and bipolar disorder), some anxiety disorders (post-traumatic stress and generalized anxiety disorder) and substance use disorders are more common in young adulthood^2^ .

It is estimated that 30% of the adults worldwide meet the diagnostic criteria for any mental disorder^3^ , and about 80% of those who suffer with mental disorders live in low- and middle-income countries^4^ . Poor mental health in youth is strongly related to other health and development problems (i.e. lower educational achievement, substance abuse, violence and poor reproductive and sexual health)^5^ , as well as higher risk of suicide^6 , 7^ . During mid-adolescence and emerging adulthood (15–29 years) suicide represents the second leading cause of death in the world, and the fourth in Brazil^8 , 9^ , and about 75% of the suicide deaths in the world occur in low- and middle-income countries^4^ .

According to data from the Brazilian Information System for Notifiable Diseases (SINAN) and the Brazilian Mortality Information System (SIM), 48,204 cases of suicide attempt and 55,649 deaths due to suicide were recorded between 2011 and 2015^9^ . About one fourth of those who attempt suicide in Brazil have already attempted suicide previously and 33.1% of women and 25.3% of men who have attempted suicide have a mental disorder^9^ . These data, though, are probably underestimated since suicide attempts are under-reported and more than 25% of suicide attempt notifications had no information about mental disorders and other health and behavioral characteristics^9^ .

Suicide behavior is a self-destructive tendency characterized by thoughts, desires and manifestations of the intention of wanting to die, planning of how, when and where to do so, and thoughts about how suicide will impact the others^10^ . Risk of suicide encompasses different risk stages, from suicidal ideation to previous attempts of suicide^10^ . Assessing the risk of suicide is often more important than seeking its cause immediately^10^ . Knowledge on the prevalence of mental disorders and suicide risk and their relationship may guide interventions that can contribute to the reduction of the burden attributed to these disorders. This study sought to (i) estimate the prevalence of some mental disorders and suicide risk in emerging adulthood (22 years) and (ii) explore the association of mental disorders with suicide risk in the 1993 Pelotas Birth Cohort.

## METHODS

### Study Setting, Design and Participants

This is a cross-sectional study using data from the 22-year follow-up of the 1993 Pelotas Birth Cohort, in Southern Brazil^11^ . In that year, all maternity hospitals in Pelotas were visited daily, and all live births (n = 5,265) from women living in the urban area were recorded (less than 1% of the deliveries were at home or other places)^12^ . Of those, 5,249 mothers consented for their children to take part in the study, and the children have undergone follow-up on numerous occasions. During childhood, only subsamples of the participants were sought^12^ . Follow-ups of the full cohort took place at 11, 15, 18 and 22 years of age^11 , 13^ . At the 22-year follow-up, 3,810 individuals were interviewed (76.3% follow-up rate)^11^ and the losses to follow-up were higher among men and at the extremes of income distribution^11^ . More detailed information about the study methodology can be found in previous publications^11^ .

### Measures

Mental disorders and suicide risk were assessed using the Mini International Neuropsychiatric Interview (MINI), version 5.0^14^ , applied by trained psychologists. MINI is a short, structured diagnostic interview validated in Brazil that explores major psychiatric disorders according to DSM-IV and ICD-10^15 , 16^ . As the interview was part of a larger follow-up assessment, including several health-related topics other than mental health, only the most prevalent and impairing disorders in this age group^2^ , with greater transcendence, were assessed: lifetime bipolar disorder type 1 (BD1), lifetime bipolar disorder type 2 (BD2), lifetime antisocial personality disorder (APD), generalized anxiety disorder (GAD, previous six months), attention-deficit/hyperactivity disorder (ADHD, previous six months), post-traumatic stress disorder (PTSD, previous month), social anxiety disorder (SAD, previous month), and major depressive disorder (MDD, previous 15 days). Definitions of mental disorders followed the DSM-5 criteria^17^ , which requires a significant impairment of symptomatic severity on individual’s life for the diagnosis of mental disorders. We investigated the level of impairment of each disorder using the following question: “Thinking about your life, school, work, home, family and friends, how much have these problems impaired you? The answer options were: no impairment (0), mild impairment (1), moderate impairment (2), or severe impairment (3). Considering the threshold for defining impairment is subjective and personal, as a conservative approach we considered impairment if the individual reported the condition caused moderate or severe impairment, as already adopted by other authors^6^ .

The MINI section about suicidality contains six questions about several components of suicide risk (wish to be dead, wish to self-harm, suicidal thoughts, suicidal planning, suicide attempt in the past month and lifetime suicide attempt). A positive answer in one of the six questions is considered current suicide risk. In our cohort study, the question about suicide attempt in the previous month was not applied. Suicide risk was based on the first four questions and lifetime attempt.

The neuropsychiatric interview was not performed in deaf and mute individuals (n = 2) and those with cognitive disabilities (n = 17), and 10 participants only answered the general interview (and not the neuropsychiatric interview), and as such being losses for this study. Moreover, 144 individuals answered the neuropsychiatric interview by phone, but the section about suicidality was not performed in these individuals since it was impossible to refer them to a health service. The final sample comprised 3,781 individuals who had complete data on mental disorders and 3,637 who had complete data on suicidality.

Confounders were assessed at the perinatal, 11- and 15-year follow-ups. At the perinatal visit, the following variables were assessed: sex (male, female), birth weight (< 2,500; 2,500–2,999, 3,000–3,499 and ≥ 3,500 grams), family income (earned by family members in the month before the interview in minimal wages, which was categorized in tertiles), maternal schooling (categorized in 0–4, 5–8, 9–11, and 12 years or more), maternal number of children (1, 2 and 3 or more), maternal marital status (with a partner, without a partner), maternal smoking during pregnancy (yes, no) and alcohol intake during pregnancy (yes, no). At the 11-year follow-up, information on maternal mental health was measured using the Self-Reporting Questionnaire (SRQ-20)^18^ . Women with a score equal to or greater than eight were classified as having common mental disorders^18^ . At the 15-year follow-up, information on participant’s skin color (categorized as white and black/brown or other) and tobacco, alcohol and illicit drug (shoemaker’s glue, solvent or thinner, cocaine, and marijuana) experimentation was assessed.

### Statistical Analysis

We assessed the prevalence of each mental disorder and suicide behavior (ideation, plan, lifetime attempt and current suicide risk) according to sex, using chi-squared test for heterogeneity to assess differences between men and women. We also described the number of disorders in the same individual and the most common co-occurring disorders. Then, we assessed the association of each mental disorder and the number of disorders with suicide risk using Poisson regression with robust adjustment of the variance, adjusted for all the covariates defined above. We found no evidence of sex differences in the associations (all *p* -values for interaction were ≥ 0.1), thus the results were not stratified by sex.

### Ethical Considerations

The 1993 Pelotas Birth Cohort study obtained ethical approval from the Medical School Ethics Committee of the Universidade Federal de Pelotas, and a full inform consent was provided by the cohort members or by their parents when individuals were younger than 18 years. Individuals with high risk of suicide were referred to health services.

## RESULTS

Table 1 shows that from the 3,781 participants included in the study, the mothers of 73.3% of the participants studied up to eight years, 40.9% were primiparous and 12.0% did not have a partner ( Table 1 ). The prevalence of maternal tobacco and alcohol use during pregnancy was 32.6% and 5.2%, respectively, and the prevalence of maternal common mental disorders was 30.0%. About half of the participants were women, 63.3% reported white skin color and 9.5% had low birth weight. The prevalence of alcohol, tobacco and illicit drug experimentation in mid-adolescence (15 years) was 59.2%, 18.2% and 1.7%, respectively.

**Table 1:** Characteristics of the participants who answered the neuropsychiatric interview at the 22-year follow-up (n = 3,781). 1993 Pelotas Birth Cohort, Brazil, 2015–2016.

Variables	n	%
Maternal characteristics		

Schooling (years)		
0–4	998	26.4
5–8	1,772	46.9
9–11	698	18.5
≥ 12	308	8.2
Family income (tertiles)		
1^st^ (lowest)	1,548	41.7
2^nd^	1,087	29.3
3^th^ (better off)	1,077	29.0
Marital status		
With partner	3,327	88.0
Without partner	454	12.0
Parity		
1	1,545	40.9
2	1,142	30.2
> 3	1,094	28.9
Smoking during pregnancy	1,234	32.6
Alcohol use during pregnancy	198	5.2
Common mental disorders	1,071	30.0

Participant’s characteristics		

Sex		
Man	1,763	46.6
Woman	2,018	53.4
Skin color		
White	2,249	63.3
Black/brown or other	1,302	36.7
Birth weight (grams)		
< 2,500	357	9.5
2,500–2,999	923	24.4
3,000–3,499	1,494	39.6
≥ 3,500	1,002	26.4
Alcohol experimentation at 15 years	2,064	59.2
Tobacco experimentation at 15 years	639	18.2
Any illicit drug experimentation at 15 years	58	1.7

**Figure 1:**
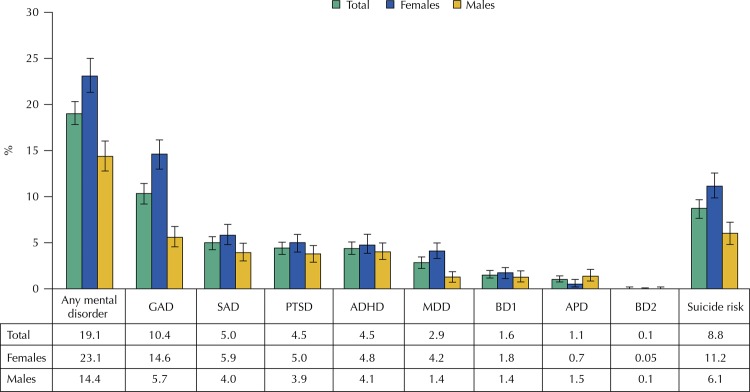
Prevalence of mental disorders at 22 years, by sex (n = 3,781). The 1993 Pelotas Birth Cohort, Brazil, 2015–2016.

GAD: generalized anxiety disorder; SAD: social anxiety disorder; PTSD: post-traumatic stress disorder; ADHD: attention deficit/hyperactivity disorder; MDD: major depressive disorder; BD1: bipolar disorder type 1; APD: antisocial personality disorder; BD2: bipolar disorder type 2.

Figure 1 shows that approximately one in five individuals had mental disorders at 22 years of age. GAD was the most prevalent disorder (10.4%, 95%CI: 9.5–11.4), followed by SAD (5.0%, 95%CI 4.3–5.7). Women had higher prevalence of GAD, SAD and MDD, whereas men had higher prevalence of APD.

Of those individuals with mental disorders, 62.8% had one mental disorder, 22.9% had two disorders and 14.3% had three or more disorders (data not shown in table). As expected, high co-occurrence of mental disorders was observed with GAD, especially among those with BD1, MDD and PTSD. Among those with BD1, 65.6% also had GAD, as shown in Table 2 .

**Table 2:** Frequency of co-occurring mental disorders at 22-years (n = 3.781). The 1993 Pelotas Birth Cohort, Brazil, 2015–2016.

Mental disorders	Co-occurring disorder (%)							

	MDD	BD1	BD2	GAD	SAD	ADHD	PTSD	APD
MDD (n = 108)	-	-	-	54.6	18.5	15.7	27.8	0.9
BD (n = 61)	-	-	-	65.6	34.4	18.0	41.0	3.3
BD2 (n = 3)	-	-	-	33.3	33.3	-	33.3	-
GAD (n = 395)	15.0	10.1	0.3	-	21.3	13.2	23.3	2.5
SAD (n = 188)	10.6	11.2	0.5	44.7	-	17.0	26.1	1.6
ADHD (n = 169)	10.1	6.5	-	30.8	18.9	-	20.7	5.3
PTSD (n = 169)	17.8	14.8	0.6	54.4	29.0	20.7	-	7.1
APD (n = 41)	2.4	4.9	-	24.4	7.3	22.0	29.3	-

MDD: major depressive disorder; BD1: bipolar disorder type 1; BD2: bipolar disorder type 2; GAD: generalized anxiety disorder; SAD: social anxiety disorder; PTSD: post-traumatic stress disorder; ADHD: attention deficit/hyperactivity disorder; APD: antisocial personality disorder.

Table 3 shows that the prevalence of suicide ideation, plan, and lifetime attempt was 2.8%, 1.7%, and 5.7%, respectively. Such behavior was higher in women. About 60% of those who had suicidal ideation also had a suicide plan, and 46.5% made a lifetime attempt of suicide. The prevalence of current suicide risk was 8.8% (95%CI 7.9–9.7) and it was also higher in women.

**Table 3:** Suicide ideation, plan and attempt, and current suicide risk at 22 years (n = 3,637). The 1993 Pelotas Birth Cohort, Brazil, 2015-2016.

Suicidality	Total %	Male	Female	p*
		
	(95%CI)	(95%CI)	(95%CI)	
In the total sample (n = 3,637)				
Ideation	2.8 (2.2–3.3)	1.8 (1.1–2.4)	3.7 (2.8–4.5)	0.001
Plan	1.7 (1.2–2.1)	1.0 (0.5–1.5)	2.2 (1.6–2.9)	0.004
Lifetime attempt	5.7 (5.0–6.5)	3.8 (2.9–4.7)	7.4 (6.2–8.5)	< 0.001
Current suicide risk	8.8 (7.9–9.7)	6.1 (4.9–7.2)	11.2 (9.8–12.6)	< 0.001
Among those with suicide ideation (n = 101)				
Plan	59.6 (49.7–69.1)	56.7 (38.4–74.9)	60.6 (48.9–72.2)	0.716
Lifetime attempt	46.5 (36.6–56.4)	46.7 (27.7–65.6)	46.5 (34.6–58.4)	0.986

* *p* -value for difference between sexes.

**Figure 2:**
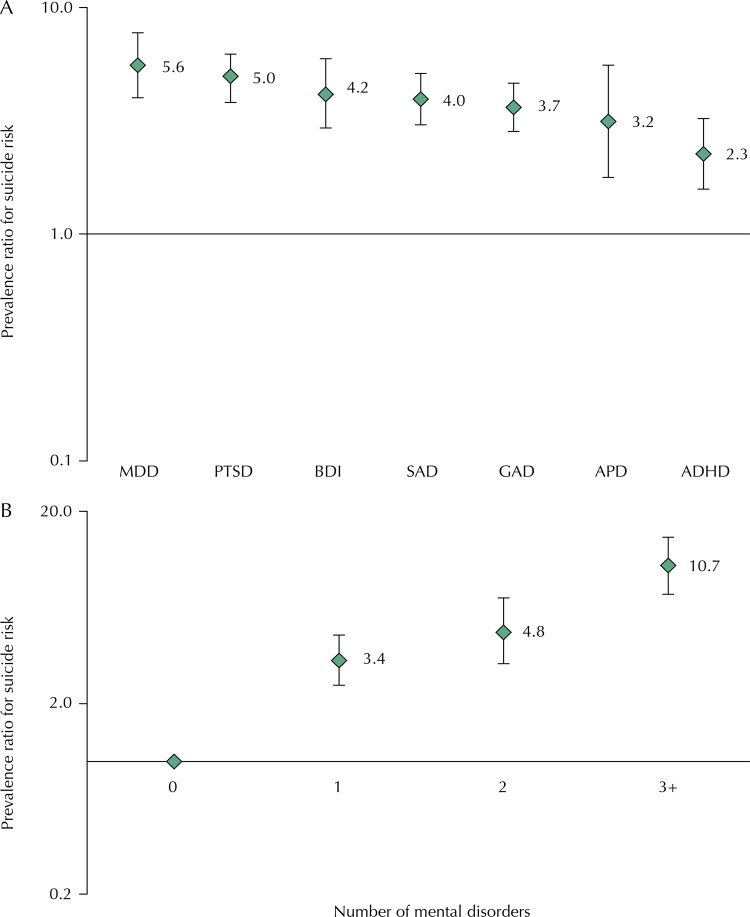
Prevalence ratio of suicide risk according to each mental disorder (A) and to the number of co-occurring disorders at 22 years (B). The 1993 Pelotas Birth Cohort.

MDD: major depressive disorder; PTSD: post-traumatic stress disorder; BD1: bipolar disorder type 1; SAD: social anxiety disorder; GAD: generalized anxiety disorder; APD: antisocial personality disorder; ADHD: attention deficit/hyperactivity disorder.

All mental disorders were associated with a higher probability of having suicide risk, and the highest risks were observed among those with MDD (PR = 5.6; 95%CI 4.1–7.8) and PTSD (PR = 5.0; 95%CI 3.9–6.3) ( Figure 2 , A). We could not assess the association between BD2 and suicide risk due to the very low prevalence of BD2 in our sample (n = 3; 0.1%). The higher the number of mental disorders, the higher the probability of having risk of suicide, and individuals with three or more disorders had 10.7-fold increase in the risk of suicide ( Figure 2 , B).

## DISCUSSION

This study showed that about 20% of young people had at least one mental disorder and 8.8% had suicide risk in early adulthood. GAD and SAD were the most prevalent disorders. Mental disorders were associated with higher probability of suicide risk, especially MDD and PTSD. The higher the number of disorders, the higher the risk of suicide.

The different methodologies (instruments used, recall period, participant’s characteristics and types of mental disorders assessed) limit the comparison of the prevalence of mental disorders across studies. A recent meta-analysis estimated a global 12-month prevalence of common mental disorders (mood disorders, anxiety disorders and substance use disorders) in adults and found a prevalence of 17.6% of any common mental disorders, which was similar in low- and middle-income countries and high-income countries, but tended to be higher in Latin America and the Caribbean (22%)^3^ . In Brazil, only few epidemiological studies estimated the prevalence of mental disorders in early adulthood^19^ , and most assessed symptoms of common mental disorders (depression and anxiety) or specific populations (students, teachers, workers)^19^ . Studies applying diagnostic interviews in general population found prevalence of mental disorders varying from 14.4%^21^ to 29.6%^20^ . The highest prevalence was found in São Paulo Megacity Mental Health Survey, carried out with adults living in the São Paulo metropolitan area, but they assessed the prevalence of a higher number of mental disorders than our study, as well as substance use disorders^20^ . Our estimates, thus, are underestimated, since other mental disorders and substance use disorders were not assessed.

We found high comorbidity between GAD and BD1 in our sample, which is similar to the results from a recent meta-analysis^22^ . Differentiating GAD and BD1 symptoms may be difficult, as their symptoms can be confused^17^ . We cannot rule out that GAD symptoms overlapped with BD symptoms^22^ , considering the DSM-5 does not require independence of symptoms to diagnose such disorders.

Data on suicide ideation, attempt and death in Brazil are obtained mainly from clinical samples or notification systems, and sub notification as well as sub register are still a problem^9 , 23^ . This study found a prevalence of 8.8% of suicide risk in youth, similar to the prevalence of 8.6% found in a previous study carried out in the same city in 2007/2008^10^ . About 60% of young people with suicide ideation in our sample had a suicide plan, and approximately 45% of those with a plan made a lifetime suicide attempt. This information is important for understanding suicidal behaviour and argues strongly for close monitoring of individuals in suicide risk, since suicide attempt is the main risk factor for suicide death^24 , 25^ . Between 30% and 60% of patients seen in emergency departments for suicide attempt have had previous attempts, and about 10% to 25% will attempt suicide again within one year^25^ .

Our results show that women have a higher prevalence of most mental disorders and suicide risk, which corroborates with previous studies^26^ . Although the exact origins of sex differences are not yet well understood, the role of sex hormones, response to stress, psychological characteristics (i.e. lower self-esteem and higher tendency for body shame and rumination in women), social differences (i.e. gender discrimination and gender inequality), and higher likelihood to experience childhood maltreatment and domestic violence are pointed out as possible explanations for the higher prevalence of mental disorders and suicide risk in women, especially of MDD and anxiety disorders. Although men have higher rates of suicide death, suicide attempts tend to be more prevalent in women^27^ , as men use more lethal methods in their suicide attempts^28^ .

All disorders assessed in this study were associated with higher prevalence of suicide risk, and the higher the number of co-occurring disorders, the higher the risk of suicide. These results are in accordance with previous results from both high-income countries and low- and middle-income countries^6 , 7 , 29 , 30^ . The association between mental disorders and suicide risk might be explained by the experience of distress or impairment in people with such disorders, as most of those who attempt suicide try to escape from intolerable distress^7 , 27^ . This may also explain the linear association found between the number of mental disorders and suicide risk due to the frequent occurrence of distress and impairment in people with multiple disorders, which may be intolerable when occurring at high levels^7^ .

Comorbidity is associated with worse prognosis of each disorder, as it increases the number and severity of complications and makes the treatment more difficult and less efficacious^31^ . According to data from the National Epidemiologic Survey on Alcohol and Related Conditions, a representative study of the United States population, the effect of mental disorders on suicide risk occurs in a general psychopathology dimension, representing the shared effects of all mental disorders, suggesting that the remission of one specific disorder may be insufficient to reduce suicide risk^30^ .

Our study contributed with data on the prevalence and relationship of mental disorders and suicide risk in Brazil, where data are still scarce and under-reported. Data on the prevalence of these disorders can help to plan policies and strategies to improve mental health. However, this study had some limitations. The main limitation of our study is the fact that information about several clinically important disorders was not included, especially other anxiety disorders and personality disorders, non-affective psychotic disorders and substance use disorders. MINI, although applied by psychologists, is a screening instrument, and does not replace the clinical diagnosis by a psychiatrist. However, when the interview is performed by well-trained health professionals, the accuracy with the clinical diagnosis by a psychiatrist is high^32^ . We could not classify the level of suicide risk, considering suicide attempt in the previous month was not assessed in our study, however we do not expect the absence of this information to have affected the prevalence of suicide risk, since those who attempted suicide in the last month were computed in our lifetime rate. The cross-sectional nature of our data does not allow to establish a causal relationship between mental disorders and suicide risk. On the other hand, recent evidence has shown that the effect of past mental disorders on the risk of suicide attempt was fully mediated by current mental disorders^30^ , suggesting that the assessment of current disorders may provide most of the information needed to assess the suicide risk associated with mental disorders. Finally, our prevalence data for mental disorders and suicide risk are restricted to youths who were born in the urban area of Pelotas, a medium-sized city with approximately 300.000 inhabitants in Southern Brazil, and therefore caution is needed regarding generalization of the findings to other regions of the country.

In conclusion, our data showed that the prevalence of mental disorders in emerging adulthood is high and about 10% of the youths have suicide risk. Co-occurrence of mental disorders was common and these disorders, individually and co-occurring, were associated with higher probability of suicide risk. Our results emphasize the importance of considering comorbidity in the study of mental health, suggesting that no only early treatment of mental disorders could help reducing the burden of these disorders, but also decreasing suicide risk.
